# MICP mediated by indigenous bacteria isolated from tailings for biocementation for reduction of wind erosion

**DOI:** 10.3389/fbioe.2024.1393334

**Published:** 2024-06-13

**Authors:** Alejandro Maureira, Manuel Zapata, Jorge Olave, David Jeison, Liey-Si Wong, Antonio Panico, Pía Hernández, Luis A. Cisternas, Mariella Rivas

**Affiliations:** ^1^ Laboratorio de Biotecnología Ambiental Aplicada BIOAL, Departamento de Biotecnología, Facultad de Ciencias del Mar y Recursos Biológicos, Universidad de Antofagasta, Antofagasta, Chile; ^2^ Escuela de Ingeniería Bioquímica, Pontificia Universidad Católica de Valparaíso, Valparaíso, Chile; ^3^ Centro Lithium I+D+i Universidad Católica del Norte, Antofagasta, Chile; ^4^ Department of Engineering, University of Campania L. Vanvitelli, Aversa, Italy; ^5^ Departamento de Ingeniería Química y Procesos de Minerales, Facultad de Ingeniería, Universidad de Antofagasta, Antofagasta, Chile

**Keywords:** MICP, mine tailings, ureolytic bacteria, biocementation, wind erosion rate

## Abstract

In this study, native ureolytic bacteria were isolated from copper tailings soils to perform microbial-induced carbonate precipitation (MICP) tests and evaluate their potential for biocement formation and their contribution to reduce the dispersion of particulate matter into the environment from tailings containing potentially toxic elements. It was possible to isolate a total of 46 bacteria; among them only three showed ureolytic activity: *Priestia megaterium* T130-1, *Paenibacillus* sp. T130-13 and *Staphylococcus* sp. T130-14. Biocement cores were made by mixing tailings with the isolated bacteria in presence of urea, resulting similar to those obtained with *Sporosarcina pasteurii* and *Bacillus subtilis* used as positive control. Indeed, XRD analysis conducted on biocement showed the presence of microcline (*B. subtilis* 17%; *P. megaterium* 11. 9%), clinochlore (*S. pasteurii*, 6.9%) and magnesiumhornblende (*Paenibacillus* sp. 17.8%; *P. megaterium* 14.6%); all these compounds were not initially present in the tailings soils. Moreover the presence of calcite (control 0.828%; *Paenibacillus* sp. 5.4%) and hematite (control 0.989%; *B. subtilis* 6.4%) was also significant unlike the untreated control. The development of biofilms containing abundant amount of Ca, C, and O on microscopic soil particles was evidenced by means of FE-SEM-EDX and XRD. Wind tunnel tests were carried out to investigate the resistance of biocement samples, accounted for a mass loss five holds lower than the control, i.e., the rate of wind erosion in the control corresponded to 82 g/m^2^h while for the biocement treated with *Paenibacillus* sp. it corresponded to only 16.371 g/m^2^h. Finally, in compression tests, the biocement samples prepared with *P. megaterium* (28.578 psi) and *Paenibacillus* sp. (28.404 psi) showed values similar to those obtained with *S. pasteurii* (27.102 psi), but significantly higher if compared to the control (15.427 psi), thus improving the compression resistance capacity of the samples by 85.2% and 84.1% with respect to the control. According to the results obtained, the biocement samples generated with the native strains showed improvements in the mechanical properties of the soil supporting them as potential candidates in applications for the stabilization of mining liabilities in open environments using bioaugmentation strategies with native strains isolated from the same mine tailing.

## Highlights

• In this study, tailings soils from a copper mine processing plant were used for the isolation of metal-tolerant ureolytic bacterial strains, resulting in a total of 46 bacterial isolates; only three of them showed urease positive activity: *Priestia megaterium* strain T130-1, *Paenibacillus* sp. Strain T130-13 and *Staphylococcus* sp. Strain T130-14.
• The initial content of CaCO3 detected by XRD in T1 tailings wall accounted for 0.828%. After biocementation tests the content increased: the biocement assay with the highest amount of calcite was that inoculated with *Paenibacillus* sp. T130-13 showing a fraction of 5.412%.
• Differences between biocemented samples and those non-biocemented (control tests) were evident in wind erosion tests and in their mechanical properties.
• In biocement samples the wind erosion rates decreased by 78%–80% compared to the control (82 g/m^2^h). *Paenibacillus* sp. T130-13 showed the lowest erosion rate with a soil loss of 16.371 g/m^2^h. For reference strains the erosion rate was 18.606 g/m^2^h and 15.640 g/m^2^h for *S. pasteurii* and *Bacillus subtilis* LN8B, respectively.

## 1 Introduction

Mine tailings (MT) are abundant worldwide and only in Chile there are approximately 740 of them (Sernageomin, 2023), mostly generated by copper mining, the country’s main mining industry. Chile is the world leader in copper production, with 5,330 million tons in 2022, representing 24.64% of world market coverage and with a projection of 6,581 million tons by 2033 (COCHILCO, 2023). The main deposits contain low grade sulfide ores, and it is estimated that in the next 10–15 years, grades between 0.5% and 0.7% will predominate (Lagos et al., 2020). After the separation of the minerals of interest (e.g., copper), approximately 97%–99% of the material extracted in a mining process will become tailings and will be discarded in areas close to the mining factory, which can be in the sea, rivers, lagoons, ravines, valleys, or in artificial structures called tailings impoundments (Ramírez, 2007; Cacciuttolo and Atencio, 2022). Considering the chemical composition, the amount and the granulometry of these tailings, they are classified as hazardous material to human health, the environment, and agricultural production. Therefore, they require specially designed containment facilities (Edrakri et al., 2014; Lagos et al., 2020; Cacciuttolo and Atencio, 2022). Studies of 540 Chilean MT showed the presence of potentially toxic elements (PTEs) such as Cu, Cr, Ni, Zn, Pb, As, Cd, Hg, and Fe; where As, Cd, Pb, and Hg being those found in the highest concentration in the same deposit, and 304 MT deposits contain at least one PTEs in concentrations higher than the hazardous threshold for the environment (Lam et al., 2020).

During the mining process, tailings slurries are transported to impoundments and as the solid fraction settles out, the remaining water is redirected back to the mining process for reuse. MT can contain ultrafine particles with a size ranging between 1 and 10 µm which makes them very susceptible to dispersion by wind, especially in arid and semi-arid areas (Wijewickreme et al., 2005; Mendez and Maier, 2008; Chen et al., 2017). Then, dust or particulate matter can reduce visibility on roads, degrade air quality in the surroundings and contaminate soils, surface waters (Blight, 2008) and in some cases marine environments when MT are located in coastal sectors (Zamarreño et al., 2020; Boada, 2021; Toro et al., 2022).

The mining industry is in constant development to reduce waste generation, improve storage as well as treatment techniques for its tailings deposits. Among them, strategies related to: (i) improvement of soil mechanical properties; (ii) chemical and physical stabilization; (iii) liquefaction, permeability and neutralization of acid leachates; (iv) resistance to water lixiviation, seismic action and wind erosion; (v) bioremediation, phytoremediation and biomineralization, are some of the most studied techniques (Edraki et al., 2014; De Giudici et al., 2019; Kiventerä et al., 2019; Barati et al., 2020; Kaseng et al., 2020; Kiventerä et al., 2020; Xie and van Zyl, 2020; East and Fernandez, 2021; Gerding et al., 2021; Rezasoltani, 2021; Woodcock, 2021; Zandarin, 2021; Jayapal et al., 2023; Si et al., 2023). Currently, biotechnological applications using biomineralizing microorganisms have generated interest in the materials industry, because they significantly reduce the energy demand to produce biocement if compared to that necessary for the conventional cement and, moreover, they can be used successfully in the neutralization of toxic elements such as PTEs or fossil fuel residues (Akyel et al., 2022). Biomineralization is defined as the production of minerals by the action of biological organisms. These minerals can be oxides, carbonates, phosphates and sulfates (Gadd, 2021). Specifically, the formation of carbonate structures mediated by biological organisms such as microorganisms, algae or mollusks is of great interest in materials science, biotechnological applications, geotechnical engineering, bioremediation, among others (Meyers et al., 2006; Zhang et al., 2023).

The most studied type of biomineralization is the microbial-induced carbonate precipitation (MICP), a biologically mediated cementation process that improves soil properties through calcite precipitation (Stocks-Fischer et al., 1999; Arias et al., 2017a) and, depending on the bacterial species, involves metabolic strategies that include photosynthesis, ureolysis, denitrification, sulfate reduction or methane oxidation (Ezzat, 2023). Ureolytic microorganisms are the most studied strains for MICP, where *S*. *pasteurii* is considered to be the reference strain, and, specifically to the research group that authored this work, the *Bacillus subtilis* LN8B (Arias et al., 2017a; Zúñiga-Barra et al., 2022). To date numerous studies have described in depth the chemical reactions that govern this process (Stocks-Fischer et al., 1999; Al-Thawadi, 2011; Arias et al., 2017b; Torres-Aravena et al., 2018; Omoregie et al., 2019), although alternative routes, such as the role of carbon anhydrase, still remain poorly understood.

Most of the studies on biocementation have been carried out in sandy soils or concrete, whereas applications in contaminated MT land are still scarce, thus representing an important scientific-technological challenge to develop (Zúñiga-Barra et al., 2022). The chemical composition of tailings is considered extreme for the development of life because they do not have abundant nutrients and there is a significant presence of PTEs, thus generating conditions hostile to be colonized by communities of microorganisms, and when microorganisms are present, a high biodiversity index has not been observed (Fan et al., 2023). Some studies in tailings soils have identified bacteria that have the ability to produce energy from inorganic electron donors for carbon fixation and tolerance mechanisms for PTEs such as the families of *Acidiferrobacteraceae*, Burkholderiaceae, Pseudomonadaceae and Gemmatimonadaceae (Sun et al., 2019). Therefore, the objective of this study has been to isolate indigenous ureolytic bacteria present in tailings soils and study their ability to biocement the tailings thanks to MICP, thus improving their mechanical properties and resistance to wind erosion.

## 2 Materials and methods

### 2.1 Characterization of sampled mine tailings

Soil samples from the copper mine tailings named T1 wall of "Las Luces” (25.40.13 S; 70.35.26 W), located south of the city of Taltal, II Region of Antofagasta, Chile, were collected. From the beginning the plant was designed to use seawater without desalination in all its mineral processes. It is located very close to the sea (approximately 7 km away) (https://www.cenizas.cl/faenas_oficinas/faena-taltal/). The theoretical chemical composition of these MT are described in Table 1 (Servicio Nacional de Geología y Minería SERNAGEOMIN, for its acronym in Spanish, national service of geology and mining, 2023) (https://www.sernageomin.cl/). The soil samples were chemically characterized in ALS Life Sciences Chile S.A. according to standard protocols. Briefly, to measure metals concentrations with acid digestion and subsequent analysis by inductively coupled plasma optic emission spectroscopy (ICP-OES); for Au determination, the reference methods Au-AA23 and Au-AA24 Fire Assay Fusion were carried out (Table 2). To obtain the mineralogical analyses two methods were used: (i) quantitative evaluation of minerals by scanning electron microscopy (QEMSCAN) analysis using TIMA technology; (ii) Bulk Mineralogical Analysis (BMA) method to identify the modal mineralogical composition with the occurrence of Cu, Fe, and S (Table 3). Finally, soil pH was determined according to the protocol described by Beretta et al. (2014). Subsequently, and prior to the tests, the tailings samples were sieved at 2 mm and then quartered to obtain 5 kg of sample for all the tests carried out in this study.

**TABLE 1:** Composition of copper tailings sediment according to SERNAGEOMIN (2023).

Major elements	Percentage (%)	Heavy metals	Quantity (g/t)	Rare earths	Quantity (g/t)
SiO_2_	52.99	**Cu**	1800	**Ce**	64.99
Al_2_O_3_	12.23	**Cr**	93	**Nd**	39.87
Fe_2_O_3_	11.26	**Ni**	67	**La**	28.18
Na_2_O	6.2	**Zn**	57	**Gd**	10.29
CaO	4.92	**Pb**	43	**Sm**	8.79
MgO	4.63	**Hg**	<0.01	**Pr**	8.55
CCP*	3.47	**As**	<20	**Dy**	7.02
TiO_2_	1.74			**Er**	5.68
K_2_O	1.04			**Yb**	4.33

**TABLE 2:** Quantification of the present concentration of elements in tailings soils by acid digestion and subsequent analysis with ICP-OES.

Parameter	Unit	DL^a^	Value
Silver (Ag)	ppm	0.01	0.55
Aluminum (Al)	%	0.01	6.83
Arsenic (As)	ppm	0.2	5.3
Barium (Ba)	ppm	10	130
Beryllium (Be)	ppm	0.005	1.33
Bismuth (Bi)	ppm	0.01	0.03
Calcium (Ca)	%	0.01	3.42
Cadmium (Cd)	ppm	0.02	0.12
Cerium (Ce)	ppm	0.01	56.9
Cobalt (Co)	ppm	0.1	25.4
Chromium (Cr)	ppm	1	27
Cesium (Cs	ppm	0.05	0.81
Copper (Cu)	ppm	0.2	1,080
Iron (Fe)	%	0.01	8.01
Gallium (Ga)	ppm	0.05	18
Germanium (Ge)	ppm	0.05	0.27
Hafnium (Hf)	ppm	0.1	6.8
Mercury (Hg)	ppm	0.005	0.064
Indium (In)	ppm	0.005	0.058
Potassium (K)	%	0.01	0.77
Lanthanum (La)	ppm	0.5	23.3
Lithium (Li)	ppm	0.2	24.3
Magnesium (Mg)	%	0.01	1.84
Manganese (Mn)	ppm	5	741
Molybdenum (Mo)	ppm	0.05	1.84
Sodium (Na)	%	0.01	5.94
Niobium (Nb)	ppm	0.1	7.7
Nickel (Ni)	ppm	0.2	13.8
Phosphorus (P)	ppm	10	1730
Lead (Pb)	ppm	0.5	30.7
Rubidium (Rb)	ppm	0.1	28.5
Rhenium (Re)	ppm	0.002	0.007
Antimony (Sb)	ppm	0.05	0.45
Scandium (Sc)	ppm	0.1	32
Selenium (Se)	ppm	1	1
Tin (Sn)	ppm	0.2	3.3
Strontium (Sr)	ppm	0.2	94.7
Tantalum (Ta)	ppm	0.05	0.54
Tellurium (Te)	ppm	0.05	0.07
Thorium (Th)	ppm	0.01	6.69
Titanium (Ti)	%	0.005	1.155
Thallium (TL)	ppm	0.02	0.08
Uranium (U)	ppm	0.1	2.2
Vanadium (V)	ppm	1	343
Tungsten (W)	ppm	0.1	0.6
Yttrium (Y)	ppm	0.1	45.3
Zinc (Zn)	ppm	2	65
Zirconium (Zr)	ppm	0.5	256
Gold (Au)	ppm	0.005–10	0.005

^a^ Detection limit.

**TABLE 3:** Modal mineralogical composition according to BMA.

Full list	Chemical composition	Percentage %
Albite	(NaCa) (AlSi_3_O_8_)	41.29
Fe Oxide	FeO	11.69
Plagioclase	NaAlSi_3_O_8_-CaAl_2_Si_2_O_8_	11.14
Anfibol	A_0-1_B_2_C_5_(Si,Al,Ti)_8_O_22_D_2_ [A = Na,K,Ca,Pb^2+^; B= Li,Na,Mg,Fe^2+^,Mn^2+^,Ca; C=Li, Na, Mg, Fe^2+^,Mn^2+^, Zn, Co, Ni, Al, Fe^3+^, Cr^3+^, Mn^3+^, V^3+^, Ti, Zr; D = OH, F,Cl,O]	11.58
Quartz	SiO_2_	7.49
Chlorite	(Mg,Fe)_3_(Si,Al)_4_O_10_	6.86
K-Feldspar	KAlSi_3_O_8_	2.42
Calcite	CaCO_3_	2.18
Ankerite	Ca(Fe,Mg,Mn) (CO_3_)_2_	1.35
Titanite	CaTiSiO_5_	1.45
Gypsum/Anhydrite	CaSO_4_	0.73
Muscovite/Sericite	KAl_2_(AlSi_3_O_10_) (F,OH)_2_	0.47
Apatite	Ca_5_(PO_4_)_3_(F,Cl,OH)	0.36
Biotite	K(Mg, Fe)_3_(AlSi_3_O_10_) (F,OH)_2_	0.19
Rutile	TiO_2_	0.14
Atacamite	Cu_2_Cl(OH)_3_	0.09
Tourmaline	(Ca,K,Na) (Al,Fe,Li,Mg,Mn)_3_(Al,Cr,Fe,V)_6_(BO_3_)_3_(Si, Al,B)_6_O_18_(OH,F)_4_	0.05
Chalcocite	Cu_2_S	0.03
Cu Oxide Minerals	CuO	0.03
Chrysocolla	(Cu,Al)_2_H_2_Si_2_O_5_(OH)_4_ x nH_2_O	0.02
Covellite	CuS	0.01
Pyrite	FeS_2_	0.01
Pyrophyllite	Al_2_Si_4_O_10_(OH)_2_	0.01
Others	-	0.42
Total		100%

### 2.2 Enrichment cultures and isolation of ureolytic bacteria

From each MT samples, a small amount of soil (1 g) was immersed in 10 mL of the following nutrient media prepared both in seawater and distilled water; Luria Broth (LB), Miller (ATCC medium: 1,065), 9 k Medium (ATCC medium: 2,436) and M9 minimal medium (M9) (ATCC Medium: 2,511). Then, the mixture soil with media was incubated at 20°C and 30°C for 96 h. Samples from MT T1, an inactive tailing that is at least 10 years old, were used, then two temperatures were used to select for mesophilic environmental bacteria that might be present in the samples. To isolate indigenous bacteria, a volume of 100 µL was extracted from each cultural broth and inoculated in solid cultures of the same composition with 1.5% w/v microbiological agar. After 24 h of incubation at 20°C and 30°C, the different colony forming units (CFU), obtained by the streak culture technique in solid nutrient medium, were determined and isolated. For subsequent assays, only the bacterial isolates were used. Since during the last years, seawater is progressively replacing freshwater in the mining industry because of water scarcity from continental sources (Fu et al., 2022), the bacterial isolates were incubated into a seawater-modified LB (LB-SW) at 20°C and 30°C for 72 h.

### 2.3 Urease activity qualitative assay

To determine the occurrence of urease activity of the bacterial isolates, the qualitative Christensen agar method (Christensen, 1946) modified (Arias et al., 2017b) was used. Briefly, a solid culture medium was prepared containing urea and seeded in depth in 2 mL Eppendorf tubes. Subsequently, the isolates were incubated at 20°C and 30°C for 48 h. Finally, a change in color from yellow to pink was considered as urease activity positive test. *S. pasteurii* and *B. subtilis* LN8B were used as positive controls for urease activity.

### 2.4 Bacterial identification by rDNA 16S, phylogenetic analysis and nucleotide sequence accession numbers

Total isolates from MT samples were phylogenetically identified by 16S ribosomal gene sequencing by using the universal primers F27 (5'-GAGAGTTTGATCMTGGCTCAG-3') and R1492 (5'-TACGGYTACCTTGTTACGACTT-3'). Isolates were grown in 5 mL of nutrient medium mixed at 125 rpm for 16 h at 20°C and 30°C. The cultures were then centrifuged at 4,500 *g* for 5 min and the cell pellet was processed using the DNeasy Power Soil Pro^®^ kit (QIAGEN cat. No. 47014) according to the manufacturer’s instructions. The PCR products were sequenced by the Sanger method at Macrogen Inc. (Santiago, Chile). Finally, the resulting sequences were analyzed using Chromas Pro software, assembled and compared with the GenBank nr database by using the BlastN Program. The sequences were uploaded to GenBank with the following correlatives PP379235, PP379236, PP379237, PP379238, PP379239, PP379240, PP379241, PP379242, PP379243, PP379244, PP379245, PP379246, PP379247 and PP379248.

### 2.5 Measurement of urease activity

Urease activity was quantified by phenol-hypochlorite assay (Natarajan 1995). Briefly, urease-positive isolates on Christensen agar were cultured and their urease activity was determined at 24, 48, and 72 h. 1 mL of culture was centrifuged at 8,000 ×g for 5 min, the supernatant was removed and 174 µL of phosphate buffer at pH 8 and 434 µL of 100 mM urea were added to the cell pellet that then were incubated at 37°C for 5 min. Subsequently, further 174 µL of phenol-nitroprusside and 174 µL of alkaline hypochlorite were added to the solution and the cells incubated at 37°C for 25 min. Finally, absorbance was determined at an optical density (OD) of 626 nm. A calibration curve was previously performed with ammonium chloride in the range of 50–500 µM. Urease activity (U/mL) is defined as 1 µM of hydrolyzed urea per minute in a volume of mL of culture and it was determined considering a 2:1 (v/v) ratio between ammonium chloride and hydrolyzed urea, where for each 1 µM of hydrolyzed urea 2 µM of ammonium chloride were generated. *S. pasteurii* and *B. subtilis* LN8B strains were used once again as positive controls.

### 2.6 Biocementation tests

For the biocementation assays, bacterial strains were previously cultured in LB-SW medium supplemented with 40% (w/v) urea and incubated at 30°C for 48 h. Then, the cultures were centrifuged at 4,500 ×g for 15 min, the supernatant was removed, and the cell pellet was re-suspended in 50 mL of fresh LB-SW medium supplemented with CaCl_2_×H_2_O and urea in four different conditions according to previous studies: i) 0.5M: 1M CaCl_2_×H_2_O: urea v/v (Chen et al., 2017); ii) 0.3M: 1M CaCl_2_×H_2_O: urea v/v (Meyers et al., 2011); iii) 1M: 0.8M CaCl_2_×H_2_O: urea v/v (Duan et al., 2021) and; iv) 0.8M: 1M CaCl_2_×H_2_O: urea v/v (this study). In parallel, the MT samples were dried by hat flow at 60°C for 72 h, with the aim of removing and/or decreasing the concentration of microorganisms naturally found in the samples. Finally, a biocement samples were built as described by Omoregie et al. (2017), mixing 150 g of mine tailings with 50 mL of bacterial culture in a conical mold (base diameter of 5 cm, height of 9 cm and top diameter of 8 cm), to be incubated in batch at 30°C for 20 days. Subsequently, 1 g of the formed biocement was used to prepare serial dilutions which were grown on Christensen agar plates to determine CFUs showing as well as not showing ureolytic activity. For CFUs with ureolytic activity, from 10 to 30 CFUs were randomly selected for phylogenetic identification by 16S ribosomal gene sequencing as described in Section 2.4.

### 2.7 Wind erosion tests in wind tunnels

**FIGURE 1:**
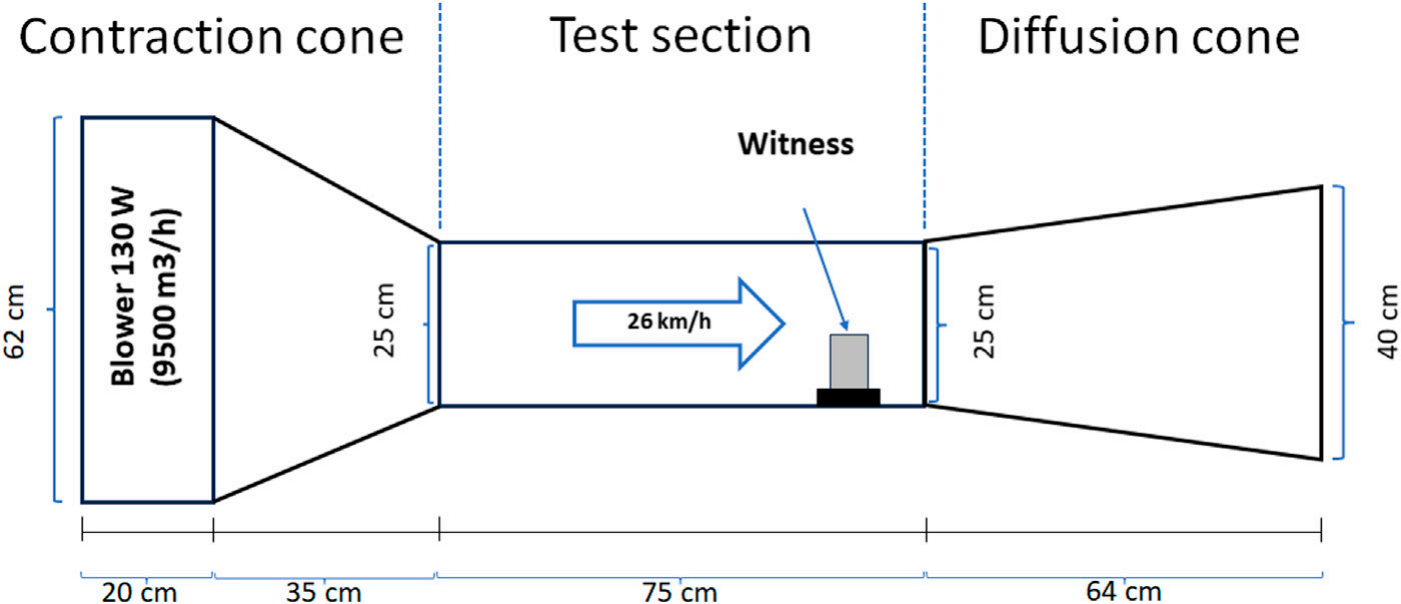
Wind tunnel design for wind erosion tests. Based on the design parameters of Moreno-Garibaldi et al. (2014).

To determine the wind erosion rate (g/m^2^h), the biocement samples were exposed to an average wind speed of 26 km/h in an open circuit subsonic wind tunnel for 45 min, considering as erosion area the sum of the upper surface and that of the face directly exposed to the wind flow. The average velocity inside the tunnel was set according to the maximum daily prevailing wind speed measured in eight points by the Cerro Moreno Antofagasta Station of the Meteorological Directorate of Chile in the Antofagasta Region, during the year 2022 (https://climatologia.meteochile.gob.cl). Subsequently, to determine the amount of mass loss, the dehydrated at 40°C for 48 h samples were weighed before and after being exposed to the wind in the wind tunnel. The wind tunnel used was designed according to the parameters described by Moreno-Garibaldi et al. (2014) (Figure 1) and validated at the Department of Mechanical Engineering of the Universidad de Antofagasta.

### 2.8 Mechanical compression tests

To determine the mechanical strength of the biocement samples, tests of 10 mm axial compression were performed by using a mechanical press (Torin, TY10003) coupled to a Dillon PAT-705 dynamometer. The pressure exerted on the surface of the samples was determined in psi.

### 2.9 Scanning electron microscopy coupled with energy dispersive X-ray (SEM-EDX) spectroscopy and XRD analysis

To determine the crystalline composition of the biocement as well as mine tailings samples before biocementation, X-ray diffraction analysis (XRD) was performed using a Bruker diffractometer model D8 Advance with DIFFRAC. EVA software and semi-quantitation of the phases with DIFFRAC. TOPAS software at the Maini Unit of the Universidad Católica del Norte. Moreover, the biocement samples were cathodically gold coated and analyzed by FE-SEM scanning electron microscopy (Hitachi, model SU5000) coupled to X-ray detection (EDX) systems (Bruker model XFlash 6,130).

## 3 Results

### 3.1 Characterization of copper mine tailings

The soil samples from the T1 tailings wall were characterized by determining the pH, whose average value was 7.11 ± 0.16. The semi-quantitative chemical composition detected by XRD analysis showed that the mine tailings contain mostly fractions of silicates (albite 74.058% and quartz 10.307%), followed by halite, iron oxides and calcite (Table 4). The presence of a significantly high amount of amorphous crystalline fraction (45.6%) in the sample did not exclude the presence of other chemical species, which were detected by complementary QEMSCAN analyses.

**TABLE 4:** Phylogenetic identification and urease activity from bacterial strains isolated from mine tailing T1.

Bacterial strain	Closest species in BLASTN^a^	E value	Similarity (%)	Identity (%)	Homolog GenBank accesión no.	Semi qualitative urease activity^b^
T20-1	*Oceanobacillus* sp. Strain JSM	0.0	99	99.19	MG893163.1	-
T20-2	*Oceanobacillus* sp. Strain JSm 1685057	0.0	98	99.53	MG893163.1	-
T20-3	*Terribacillus halophilus* isolate T-h1	0.0	98	99.53	LK054639.1	-
T130-001	*Priestia megaterium*	0.0	100	99.73	MH071287.1	++
T130-006	*Oceanobacillus* sp. Strain JSM 1685057	0.0	99	99.80	MG893163.1	-
T130-007	*Terribacillus aidingensis*	0.0	72	95.48	MG008671.1	-
T130-008	*Bacillus* sp. 64	0.0	99	99.53	GQ249102.1	-
T130-009	*Terribacillus aidingensis* strain DMT04	0.0	99	99.91	CP077639.1	-
T130-011	*Kocuria* sp. CNJ787 PL04	0.0	99	99.45	DQ448773.1	-
T130-013	*Paenibacillus* sp. D3	0.0	99	99.53	JQ345703.1	++
T130-014	*Staphylococcus* sp	0.0	98	98.69	OQ923815.1	++
T130-015	*Bacillus megaterium* strain MBFF6	0.0	99	99.73	HQ840732.1	-
T130-019	*Bacillus* sp. MB-7	0.0	98	99.53	AF326364.1	-
T130-026	*Bacillus cereus* strain 1	0.0	99	99.73	FJ435213.1	-
T130-030	*Bacillus* sp. Strain XIXJ042	0.0	99	98.71	MH801102.1	-
T130-032	*Bacillus paramycoides* strain SrAM4	0.0	99	99.08	MT066092.1	-
T30-27	*Virgibacillus halodenitrificans* strain PDB-F2	0.0	98	99.66	CP017962.1	-
T30-28	*Virgibacillus halodenitrificans* strain SQA-2	0.0	96	78.50	MT114582.1	-
T30-29	*Virgibacillus halodenitrificans* strain ARSS13	0.0	98	99.73	MT317192.1	-
T30-31	*Bacillus cereus* strain 1	0.0	99	99.80	FJ35213.1	-
M9FZ	*Mesobacillus subterraneus* strain A8	0.0	99	99.29	KY202701.1	-
9k3	*Bacillus* sp. W1	0.0	98	100	KT444619.1	-
M9C2	*Mesobacillus subterraneus* strain A8	0.0	99	99.03	KY202701.1	-

^a^ Sequence homology was determined with BLASTN.

^b^ Activity expression in Christensen urea agar plates at 24 h. Positive: ++; Negative: -.

Table 3 shows the predominant mineralogical species present in the MT samples which are mainly albite, ferric oxides, quartz, and clays. The presence of carbonates is also found. The ICP-OES analysis resulted in the presence of different elements, including PTEs in different concentrations: Fe (8.01%), Al (6.83%), Na (5.94%), Ca (3.42%), Ti (1.155%), Mg (1.84%), P (1,730 ppm), Cu (1,080 ppm), followed by Mn (741 ppm), V (343 ppm), Zr (256 ppm), Ba (130 ppm), Sr (94.7 ppm), etc .,… (see Table 2 for a detailed list). These results show similarities with the theoretical composition described by SERNAGEOMIN (Table 1), but this method did not detect the presence of rare earths.

### 3.2 Selection of ureolytic bacterial strains

Forty-six bacterial strains were isolated from mine tailings in enrichment solutions. In LB-FW, 11 isolates were obtained at 20°C and 32 isolates incubated at 30°C. Two strains were also isolated from M9 medium in FW at 20°C and one from 9K-FW at 30°C. Sepúlveda et al. (2021) isolated 22 strains from mine tailings. In addition, growth in the different culture media qualitatively demonstrates the presence of acidophilic bacteria (9K medium), chemoautotrophs (M9 medium) and, to a greater extent, heterotrophs (LB medium). Subsequently, after immersing the isolates into LB in seawater, 23 strains were selected that were able to grow under these conditions (Table 5). During the Christensen assay, only three of the isolates showed a positive urease activity (T130-01, T130-13 and T130-14) (Table 4). The rest of the bacteria did not show any change in their color, thus showing a negative urease activity.

**TABLE 5:** Crystalline composition of tailings sediment T1 before and after treatments with *P. megaterium* T130-1, *Paenibacillus* sp. T130-13 and *Staphylococcus* sp. T130-14 strains.

Crystalline phase	Chemical composition	Tailing control T1 (%)	*S. pasteurii* ^a^	*B. subtilis* LN8B^a^	*P. megaterium* T130-1^a^ (%)	*Paenibacillus* sp. T130-13^a^ (%)	*Staphylococcus* sp. T130-14^a^ (%)
Quartz	SiO_2_	10.307	19.2	21.3	20.4	17.3	22.0
Albite	(NaCa) (AlSi_3_O_8_)	74.058	39.7	36.4	30.4	41.8	39.2
Magnetite	Fe^+2^Fe_2_^+3^O_4_	1.287	1.0	3.4	2.8	2.0	2.0
Halite	NaCl	4.244	4.0	1.7	2.8	0.7	2.8
Calcite	CaCO_3_	0.828	6.6	3.2	3.2	5.4	1.8
Microcline	KAlSi_3_O_8_	-	9.1	17.0	11.9	10.3	8.0
Hematite	Fe_2_O_3_	0.989	5.2	6.4	4.5	1.7	5.9
Clinochlore	(MgFeAl)_6_(SiAl)_4_O_10_(OH)_8_	-	6.9	5.8	3.3	3.3	6.2
Magnesiumhornblende	NaKCa_2_Mg_3_FeTiAl	-	8.3	4.8	14.6	17.8	12.2
Amorphous	(AlSi_6_O_23_(OH))	45.6	55.6	62.0	62.9	55.3	56.4

^a^ Samples extracted from biocement cores.

Phylogenetic identification by 16S ribosomal RNA gene sequencing determined the presence of nine bacterial genera (Table 5), where the genus *Bacillus* predominates (35%), followed by *Oceanobacillus* (13%), *Terribacillus* (13%), *Virgibacillus* (13%), *Staphylococcus* (13%), *Mesobacillus* (9%), *Priestia* (4%), *Kocuria* (4%) and *Paenibacillus* (4%). The bacteria that tested positively for urease activity were *Priestia megaterium* strain T130-1, *Paenibacillus* sp. Strain T130-13 and *Staphylococcus* sp. Strain T130-14, globally representing 13% of the total obtained isolates. These isolates were then characterized as Gram-positive bacteria with absence of hemolytic activity on blood agar plates (data not shown).

### 3.3 Characterization and ureolytic activity of the isolates and biocementation tests

**FIGURE 2:**
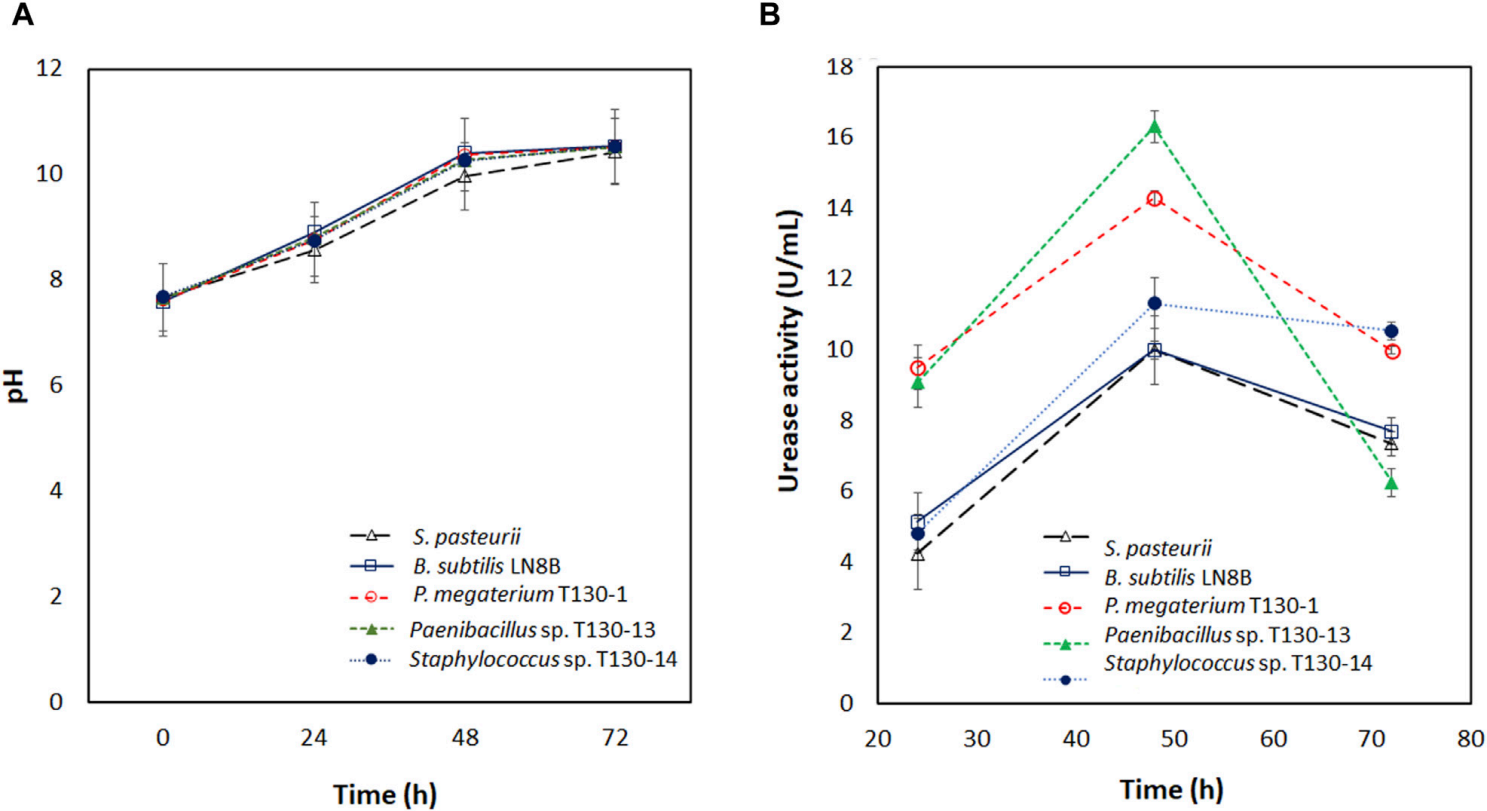
**(A)** pH of the bacterial cultures at 30°C incubated for 72 h; **(B)** Urease activity in U/mL for bacterial cultures at 30°C for 72 h ● corresponds to *S. pasteurii*; ○ corresponds to *Bacillus subtilis* LN8B; ▼ corresponds to *P. megaterium* T130-1; D *Paenibacillus* sp. Corresponds to T130-13; ■ corresponds to *Staphylococcus* sp. T130-14.

The pH values measured during the incubation of the strains (Figure 2A) show that at 24 h the cultures reach the ranges described for the activation of the urease enzyme, which occurs between pH six and 9 (Stocks-Fischer et al., 1999). In *P. megaterium* bacteria, the highest activity of this enzyme in biocementation assays is achieved between pH 7.5 to 8.5 (Sun et al., 2019). During 48 and 72 h the pH values increased to a range between 9.97 and 10.54. This increase was due to the urea hydrolysis and caused a decrease in urease activity, as demonstrated by the results of Stocks-Fischer et al. (1999) in *Bacillus pasteurii*, with a decrease in its urease activity by 50% approximately at pH close to 10. This result is similar to that obtained in this study.

For the isolated strains, urease activity (U/mL) was found to be similar or significantly higher in all the strains compared to the reference bacteria *S. pasteurii* and *B. subtilis* LN8B (Figure 2B). *P. megaterium* strain T130-1 and *Paenibacillus* sp. Strain T130-13 showed a bell-shaped behavior profile; at 24 h they reached values ranging from 9 to 10 U/mL, whereas at 48 h, from 14 to 16 U/mL and finally at 72 h, there has been a decrease since 10 to 6 U/mL. On the other side, *Staphylococcus* sp. Strain T130-14 at 24 h showed an enzymatic activity of 5 U/mL, twice lower than the other two cultures, with no significant difference with the reference bacteria. Subsequently, at 48 h it increased its activity to 11 U/mL and maintained it until 72 h. This increase was significantly higher than that of the reference bacteria.

**FIGURE 3:**
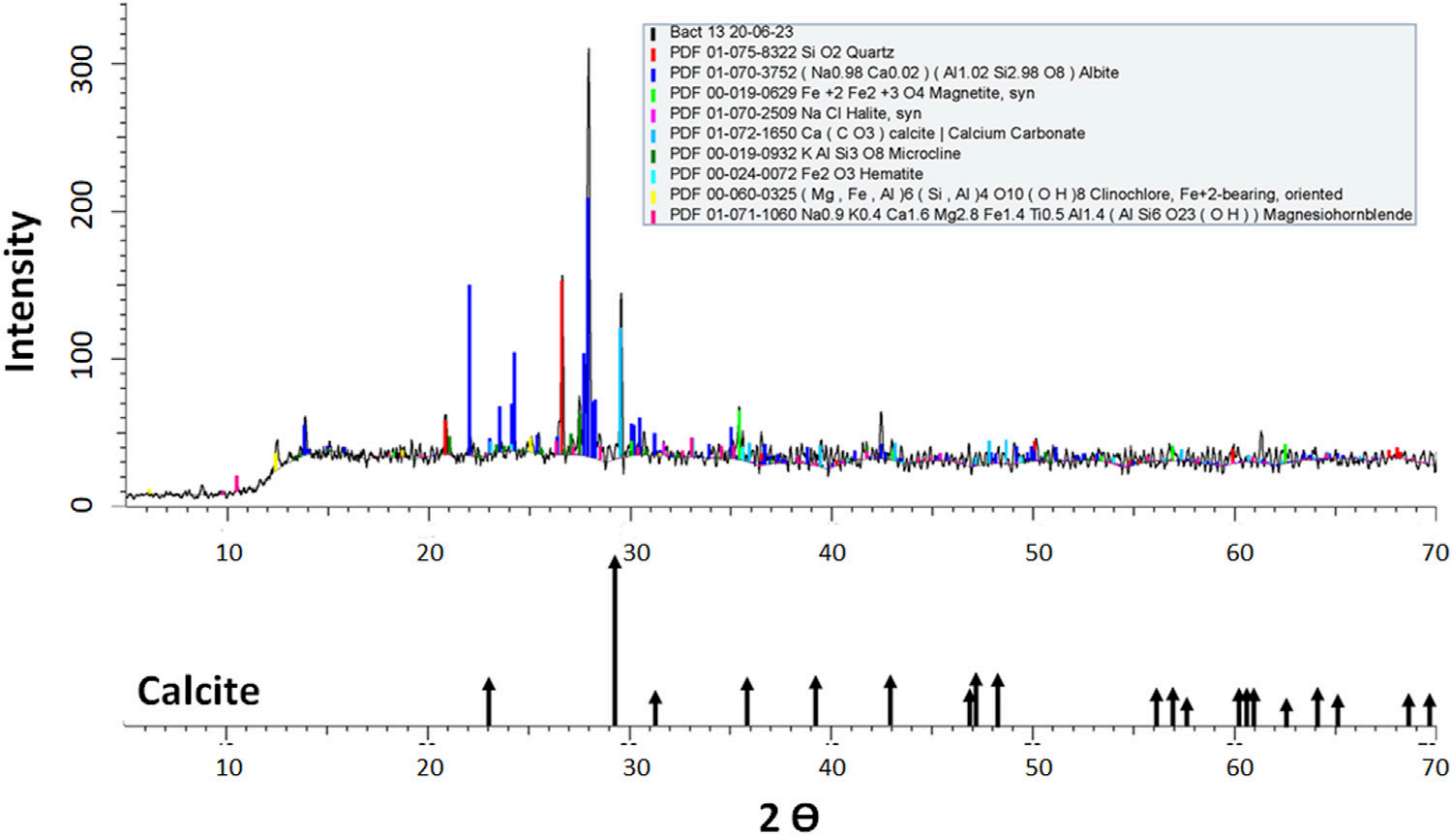
X-ray diffractogram of sediment extracted from the *Staphylococcus* sp. T130-14 biocement core.

XRD analysis of samples from T1 tailing wall and biocement samples showed the presence of quartz, albite, magnetite, halite, calcite, microcline, hematite, clinochlore and hornblende crystals (Table 5); the most abundant minerals were albite and quartz. However, it should be considered that other crystalline forms may exist and their quantification may not be reliable due to the high percentage of amorphous crystalline fraction present in all the samples (between 45.6% and 62.9%) (Table 5). Regarding the presence of calcium carbonate detected by XRD, the results are in accordance with the diffraction profile for calcite (Figure 3) of the T1 tailings wall, showing a content of 0.828% before the biocementation test. Subsequently, after biocementation test conducted with the bacterial strains, the biocement assay with the highest amount of calcite was that inoculated with *Paenibacillus* sp. T130-13 with a fraction of 5.412%, followed by that inoculated with *P. megaterium* T130-1 with a fraction of 3.191% and finally that inoculated with *Staphylococcus* sp. T130-14 with a fraction of 1.810% (Table 5). In all the biocementation tests with the ureolytic strains, the amount of calcite was higher than that present in the untreated MT samples according to XRD analysis in Table 5, however, according to modal mineralogical composition by BMA analysis (Table 3) calcite concentration amounted to 2.18% and only strains T130–13 and T130-1 exceeded this value.

### 3.4. Biocement analysis

**FIGURE 4:**
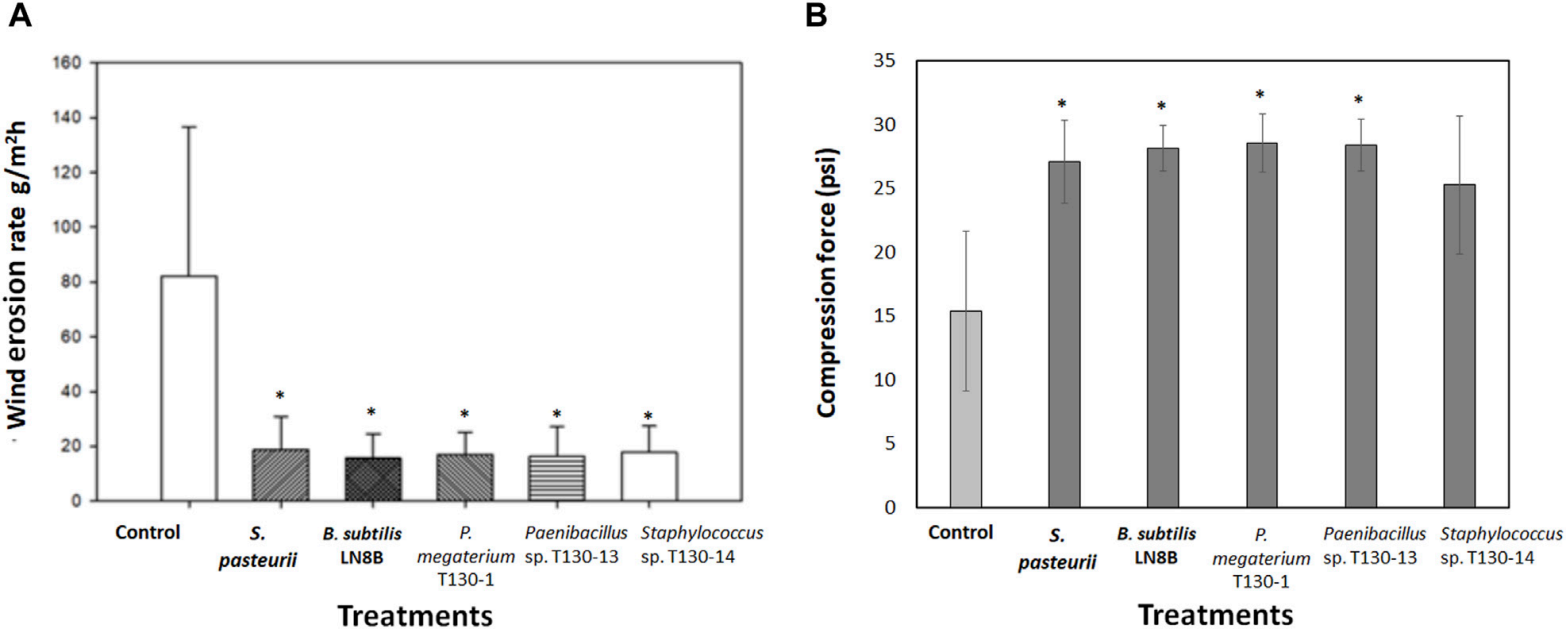
Results of wind erosion rate at 26 km/h in tailings biocement cores **(A)**; Compression force exerted on tailings biocement cores **(B)**. Treatments: Control without bacterial strains; *S. pasteurii*; *Bacillus subtilis* LN8B; *P. megaterium* strain T130-1; *Paenibacillus* sp. Strain T130-13; and *Staphyloccocus* sp. Strain T130-14. (*) indicates significant difference with respect to the control *p* < 0.05.

**FIGURE 5:**
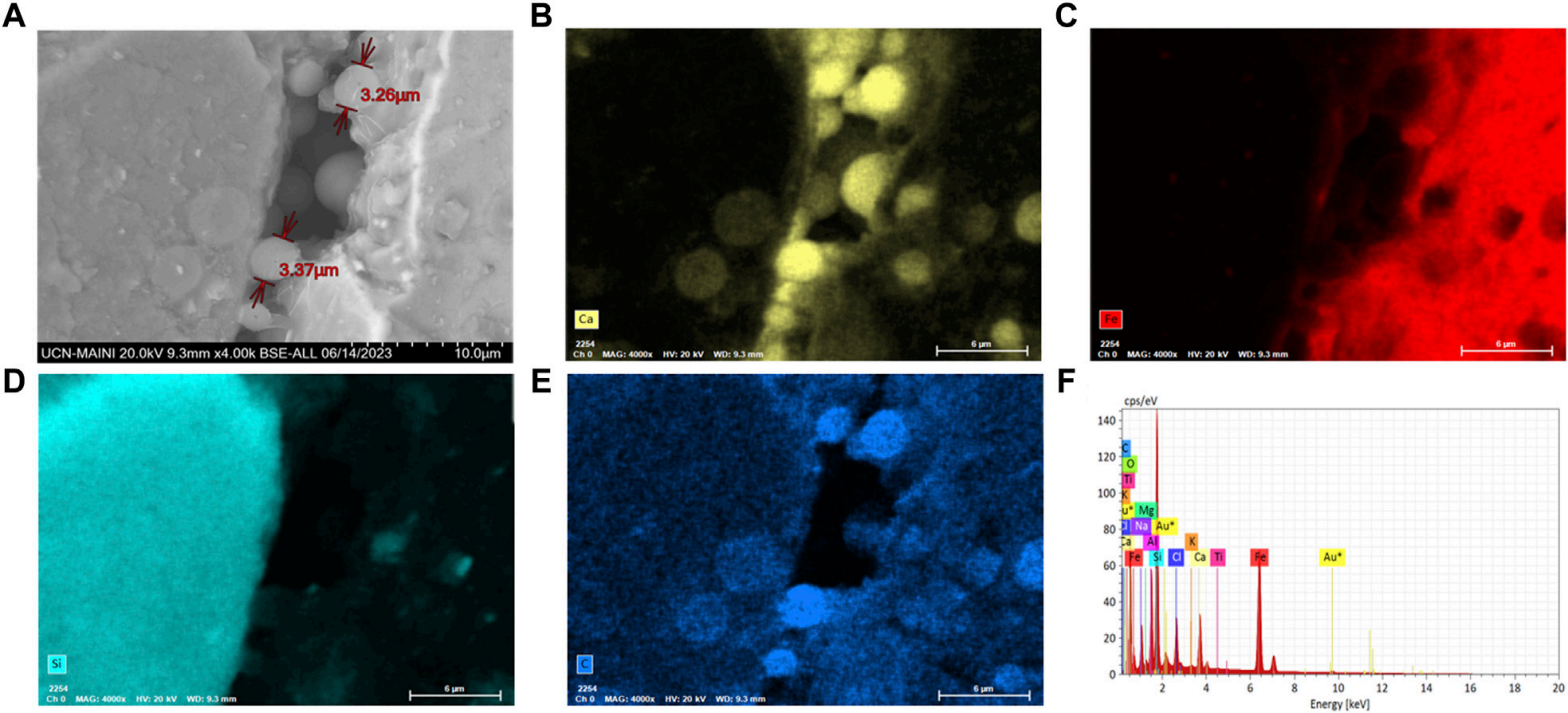
**(A)** FE-SEM-EDX scanning electron microscopy of *Staphylococcus* sp. T130-14 bacteria colonizing micropores in tailings sediment; **(B)** Calcium (yellow); **(C)** iron (red); **(D)** silica (blue-green); and **(E)** carbon (blue); **(F)** Chemical mapping with FE-SEM-EDX.

The results obtained in the wind tunnel showed that in the biocement samples the wind erosion decreased by 78%–80% compared to the control sample (82 g/m^2^h) (Figure 4A). The biocementation with *Paenibacillus* sp. T130-13 resulted in the lowest erosion rate with a soil loss of 16.371 g/m^2^h, whereas the biocementation with *P. megaterium* T130-1 and *Staphylococcus* sp. T130-14 resulted in similar mass losses of 16.371 g/m^2^h and 17.739 g/m^2^h. Such erosion rates were very close to those achieved testing biocement obtained with the reference strains (*S. pasteurii* 18.606 g/m^2^h and *B. subtilis* LN8B 15.640 g/m^2^h). Then, the compression results (Figure 4B) showed that the biocement with *P. megaterium* T130-1 (28.578 psi) and *Paenibacillus* sp. T130-13 (28.404 psi) required higher pressure than the control (15.427 psi), thus improving the compressive strength capacity by 85.2% and 84.1% compared to the control. It is worthy to state that at the end of biocementation tests counts of the CFUs formed (Figure 5) were performed and molecular identification carried out by analyzing their 16S ribosomal gene. The relative results confirmed the presence of bacteria originally added in each biocementation test (data not shown). It was possible to isolate a significantly higher number of CFUs with ureolytic activity in the biocement formed with *Paenibacillus* sp. T130-13 (51 CFU/mL ureolytic), on the contrary to the lower number of CFUs obtained in the biocement formed with *S. pasteurii* (4 CFU/mL ureolytic), *P. megaterium* T130-1 (4.3 CFU/mL ureolytic) and negative control (10.3 CFU/mL ureolytic) (Figure 5).

**FIGURE 6:**
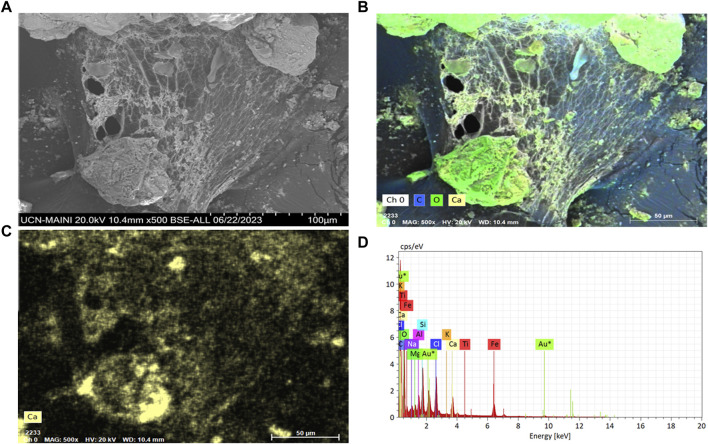
**(A)** Scanning electron microscopy in FE-SEM-EDX of biofilms of the bacterium *P. megaterium* strain T130-1 in sediment of the biocement test; **(B)** carbon (blue) and oxygen (green); **(C)** Calcium (yellow); and **(D)** Chemical mapping with FE-SEM-EDX.

**FIGURE 7:**
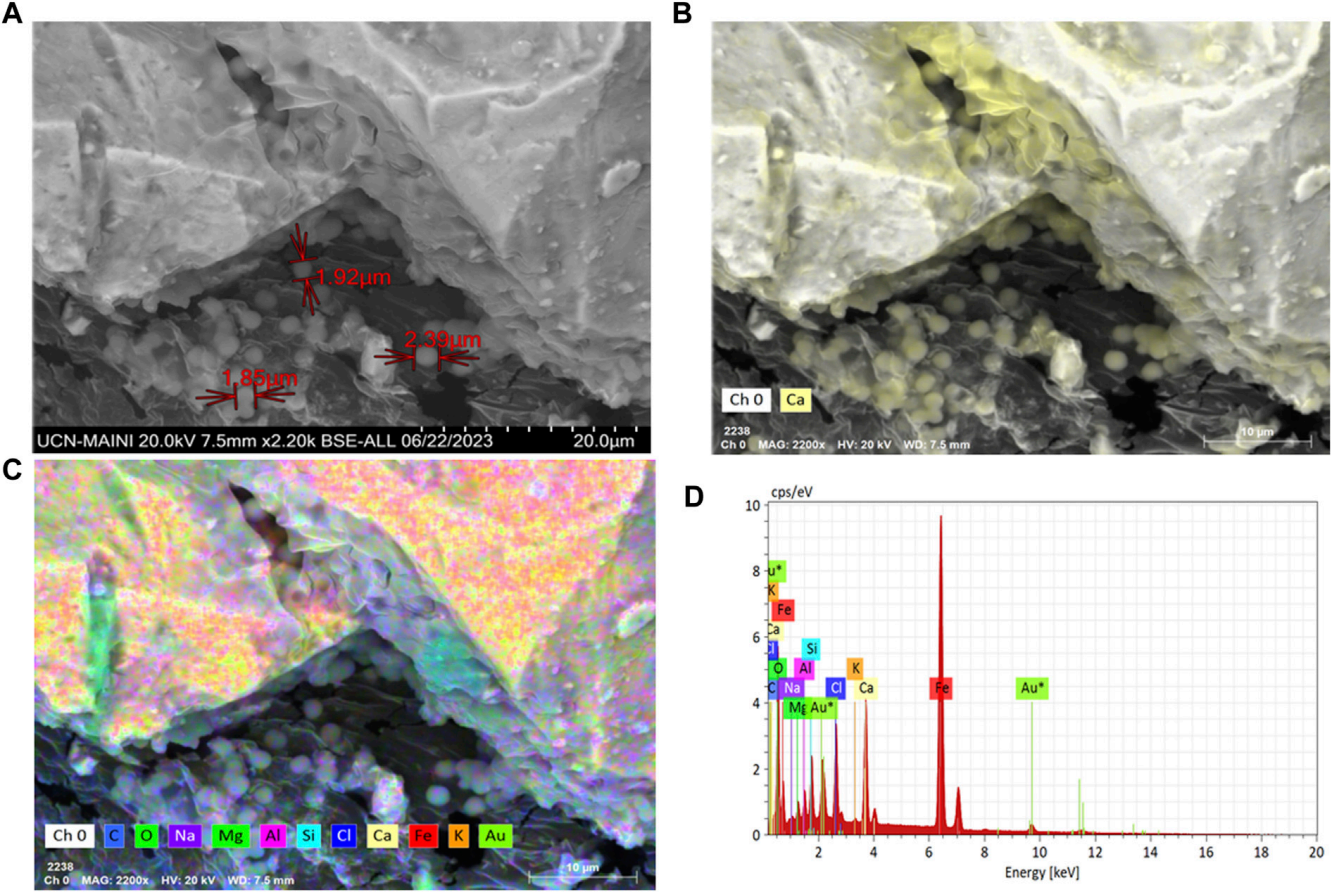
**(A)** Scanning electron microscopy in FE-SEM-EDX of *S. pasteurii* biofilms on biocement; **(B)** Carbon (blue), Oxygen (green); **(C)** Calcium (yellow) FE-SEM image; and **(D)** chemical mapping with FE-SEM-EDX for calcium.

**FIGURE 8:**
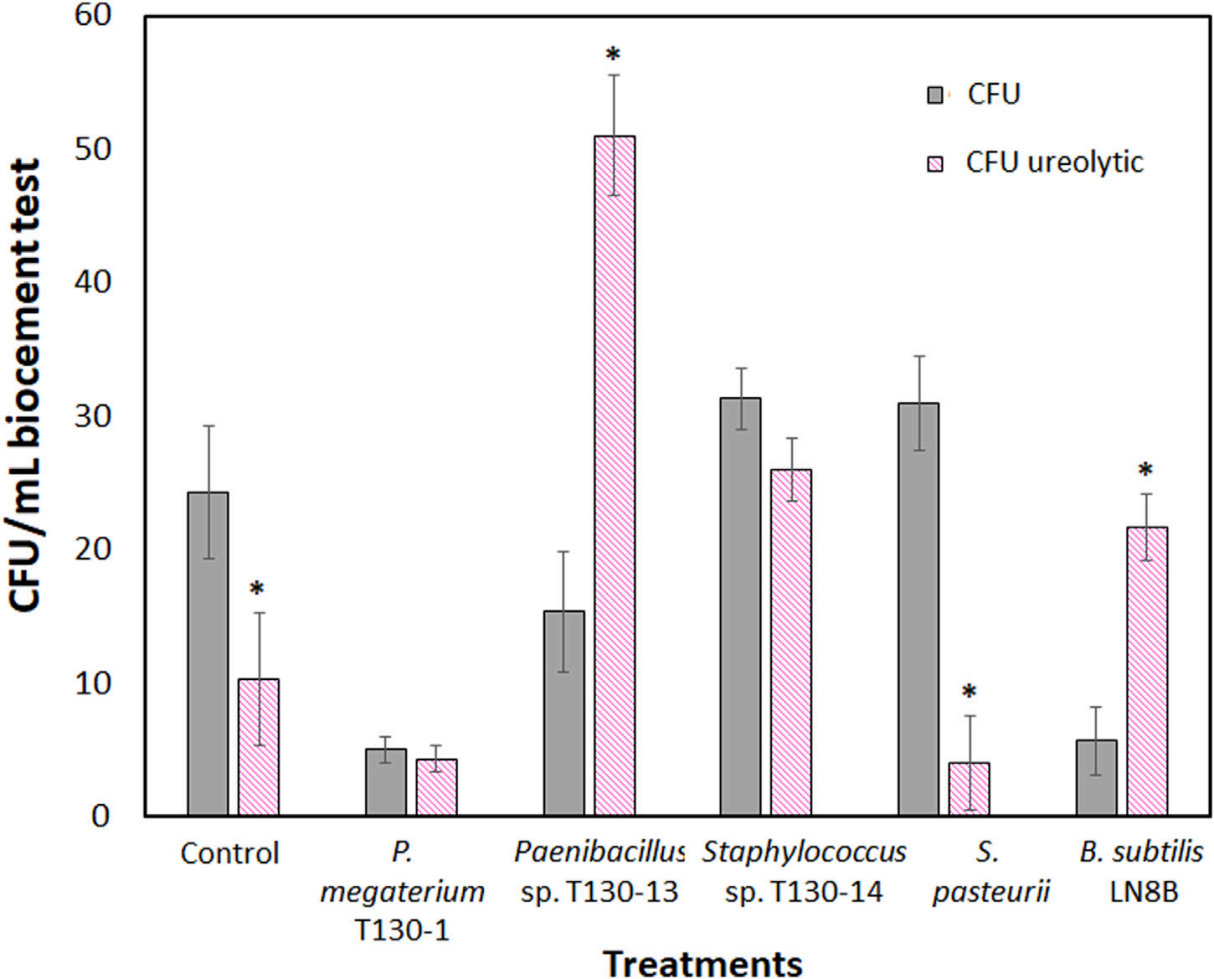
Colony forming units (CFU) isolated from biocementation test core after 30 days of treatment. Ureolytic activity was determined by positive activity for each CFU on Christensen agar plates.

Particle adhesion and agglomeration were observed in all the biocement samples where *S. pasteurii* and the native ureolytic strains were used (Figures 6–8). Biofilm-like structures were found to be present in cracks and spaces between particles. Particles ranging from 3 to 15 µm in size forming larger agglomerations with abundant calcium, carbon and oxygen (Figure 7C) were also observed. These structures evidenced the adhesion of the particles due to the colonization of bacteria and the formation of calcium carbonate.

## 4 Discussion

### 4.1 Characterization of copper mine tailings

The MT samples were mainly composed of feldspar (albite, plagioclase, K-feldspar) and silicates. The amount of sulfurous compounds was low, only covellite and pyrite were identified. Therefore, the probability of generating acid leachate is low. Although the pH of MT was adequate for the growth of bacteria, the variety and concentration of different PTEs were responsible for a harsh environment to an abundant growth of microorganisms.

### 4.2 Selection of ureolytic bacterial strains

Microbial growth in the various soil media can be interpreted as an indicator of the biological functions performed by the bacteria and the recovery status of the soil. According to Sun et al. (2019) the recovery of mine tailings begins with the action of "Keystone” microorganisms that have the ability to obtain energy from inorganic sources, thus generating: (i) the modification in the medium such as the annulment of PTEs toxicity; (ii) the stabilization of pH; and (iii) the increase of the availability of carbon sources for the subsequent colonization of heterotrophic organisms. According to the above considerations, the results obtained in this work hint at the presence of heterotrophic organisms capable of obtaining energy from organic sources, by hydrolyzing urea, since this molecule has an organic origin. The low vitality of the other isolates may be due to stress generated by the high availability of carbon sources as described for chemoautotrophic bacteria, such as *Acidithiobacillus ferrooxidans* ATCC 23270 and/or sensitivity to pH changes (Zhou et al., 2012; Falagán et al., 2019). Another possible reason of this phenomenon could have been the osmotic stress due to the increased salinity caused by seawater that prevented the proliferation of the isolated strains (Navada et al., 2020).

Table 5 shows that the presence of ureolytic microorganisms in soils contaminated with PTEs cannot be considered a coincidence. In a metagenomic study on mine tailings, 511 ureolytic species were identified and only 464 of them could tolerate the presence of PTEs (Hu et al., 2021). Although the isolation of native ureolytic strains in mine tailings has been successfully performed in several cases, it has not been possible to isolate an abundant number of strains. This is demonstrated in studies conducted on mine tailings in Iran, where 47 out of 76 isolates showed a positive urease activity (Jalilvand et al., 2019). Other results like those obtained in this study were obtained from copper tailings with the isolation of 22 strains and from copper-nickel mining wastes with the isolation of only two strains, thus proving the difficulty in obtaining an abundant number of ureolytic bacterial strains from mine tailings (Sepúlveda et al., 2021; He et al., 2022). On the other hand, some studies reported that native species were isolated from mine tailings and used for biocementation such as *Lysinibacillus fusiformis*, *Bacillus firmus*, *Variovorax boronicumulans*, *Stenotrophomonas rhizophila*, *Bacillus* sp. And *Staphyloccocus equorum* (Yang et al., 2016; Jalilvand et al., 2019; Sepúlveda et al., 2021; He et al., 2022; Liu et al., 2023).

The ureolytic bacteria *P. megaterium* strain T130-1, *Paenibacillus* sp. Strain T130-13 and *Staphylococcus* sp. Strain T130-14 represents a fraction of 13% of the total isolates obtained. No biocementation processes in mine tailings have been described using *Paenibacillus* sp. However, in its genome, genes for the alpha (*ureC*), beta (*ureB*) and gamma (*ureA*) subunits of the urease enzyme complex are encoded, in addition to other subsets of accessory genes such as *ureEFD* and *ureFDG* (Hoke et al., 2021). On the other hand, in *Paenibacillus mucilaginosus*, calcium and calcite biomineralization studies were conducted in industrial soils (Zhan et al., 2016) and the presence of *Paenibacillus* has been described in soils contaminated with arsenic, antimony tailings, lignite mine wastes, abandoned lead-silver-zinc mining waste and around mine tailings with the presence of PTEs, thus demonstrating the ability of this group of microorganisms to tolerate these contaminants (Shagol et al., 2014; Xiao et al., 2019; Karuriya and Choudhary, 2022; Nosalova et al., 2022; Wang et al., 2022).

Formerly classified as *Bacillus megaterium*, the bacterium *P*. *megaterium* has been studied extensively for its resistance to PTEs and its contribute in the improvement of contaminated soils for plant settlement, including trials on abandoned mine tailings (Bennis et al., 2022; Lin et al., 2022; Nie et al., 2023; Pattanaik et al., 2023). Guzman-Moreno et al. (2022) found the presence of all the genes for urease synthesis (*ureA*, *ureB*, *ureC*, *ureE*, *ureF* and *ureD*), however, urease assays resulted negative, suggesting that the expression of this enzyme could be induced only in presence of urea. The result above is in accordance with studies where carbonate precipitation, improvement of concrete properties and induction of calcium carbonate precipitates in mine tailings have been achieved using this bacterium (Lian et al., 2006; Chaurasia et al., 2019; Kim and Lee, 2019; Nasser et al., 2022). Whereas, *Staphyloccocus* sp., is mainly known for its negative interaction with humans, being this strain responsible for various diseases caused by its infection (Bore et al., 2007). However, this genus of bacteria is widely distributed in soils contaminated with different PTEs such as Cu, Pb, Cr, Zn, As and Hg. These bacteria are supposed to play a role in soil remediation as they enhance plant settlement in contaminated areas (Domingues et al., 2020; Fashola et al., 2020; Narayanan et al., 2020; Nosalova et al., 2022; El-Imam et al., 2023; Rojas-Solis et al., 2023; Xia et al., 2023). Like the other selected bacteria, it presents in its genome all the genes for urease synthesis (*ureA*, *ureB*, *ureC*, *ureE*, *ureF* and *ureD*), but unlike *P. megaterium* the urease activity in this bacterium is constitutive and when induced, its urease activity increases by 50% (Oki et al., 2010). This bacterium has been found to exhibit the ability of biocementing sand soils by precipitation of calcium carbonate and also mitigating soil liquefaction (Moosazadeh et al., 2019; Kalantary et al., 2022). Specifically, in copper mine tailings, bacteria of this genus have been used for testing co-precipitation of PTEs with calcium carbonates (Sepúlveda et al., 2021).

### 4.3 Characterization and ureolytic activity of the isolates and biocementation tests

Ureolytic bacteria isolated from copper mine tailings showed significantly higher urease activity than *S. pasteurii* and *B. subtilis* LN8B (Figures 2A, B). *P. megaterium* strain T130-1 and *Paenibacillus* sp. Strain T130-13 presented their highest activity at 48 h, followed by a declining phase until 72 h. This trend was also observed in Oki et al. (2010) for *P. megaterium*, thus demonstrating that these bacteria present a constitutive urease activity and, when stimulated with urea, they can increase their urease activity by 50%. In contrast, *Staphylococcus* sp. Strain T130-14 shows a higher and sustained urease activity of 11 U/mL up to 72 h of culture. This behavior is similar to that described by Sepúlveda et al. (2021) for *S. equorum*. This trend may be related to the non-constitutive expression of urease in ureolytic bacteria as demonstrated by Guzmán-Moreno et al. (2022) in *B*. *megaterium*, where the bacterium presents in its genome all the genes for urease synthesis, but in biochemical tests did not show a positive urease activity.

After the biocementation tests, XRD showed the presence of quartz, albite, magnetite, halite, calcite, microcline, hematite, clinochlore and hornblende crystals (Table 4); the most abundant structures were albite and quartz compared to the control. In all the biocementation tests with the ureolytic strains the amount of calcite was higher than those present in the untreated mine tailings, thus proving the ability to induce calcium carbonate precipitation owned by the native strains in calcite crystalline form despite the presence of PTEs and other compounds in the T1 tailings wall. Such results are in accordance with those obtained from other MICP studies, where ureolytic bacteria in gold and copper mine tailings promoted the precipitation of calcite crystals, vaterite or aragonite (Yang et al., 2016; Kim and Lee, 2019) and with those where bioimmobilization and biomineralization assays of PTEs were conducted by MICP and the calcite formation was found (Yang et al., 2016; Sepúlveda et al., 2021; Yin et al., 2021).

### 4.4 Biocement analysis

The biocement obtained with ureolytic bacteria showed a wind erosion rate lower than control (no bacteria) test by 78% and 80% (Figure 4A). The lowest erosion rate was obtained in the biocementation with *Paenibacillus* sp. Strain T130-12. These results are similar to those obtained by Zhu et al. (2020) that conducted wind erosion tests on bio-stabilized coal mine tailings with *S. pasteurii* and found that the wind erosion rate decreased by 58.4% (24.57 g/m^2^h) compared to the control test (59 g/m^2^h). On the other hand, in sandy soils, biostabilized with *S. pasteurii* under the wind speeds ranging between 35 and 55 km/h in a wind tunnel, the decrease in mass losses resulted ranging between 99.5% and 96.5% (Maleki et al., 2016).

Additionally, results from the compression tests (Figure 4B) showed that the *P. megaterium* T130-1 and *Paenibacillus* sp. T130-13 biocement required higher pressure than the control test, thus increasing the compressive strength capacity by 85.2% and 84.1% compared to the control test. Similar results were obtained with *S. pasteurii* (27.102 psi) and *B. subtilis* LN8B (28.144 psi), showing an improvement in compressive strength by 75.7% and 82.4%. Using *Staphylococcus* sp. T130-14 (25.277 psi), the compressive strength was increased by 63.9%, without being statistically significant. The increase in mechanical compressive strength can be interpreted as a greater capacity to withstand soil loads and pressures (Bowles, 1997). This behavior may be due to the increased compaction of the soil particles by the precipitation of calcium carbonate between the pores and internal spaces of the soil, thus increasing the cohesion of the particles. These results indicate that the biocemented material increases its capacity to resist to mass loss by wind action, as occurs in agricultural soils where it is described that a great compaction and the presence of conglomerates decrease mass loss by wind erosion (Calderón Condori, 2017).

The mechanical strength values obtained were low compared to those described in biocement using the native strain *L. fusiformis* that reached pressures up to 50 psi (He et al., 2022). The value above hints at the possibility of using successfully native strains for the biocementation of mine tailings and optimize the technique for tailings stabilization as described by Buikema et al. (2017) that conducted deformation resistance tests using native strains and *S. pasteurii* in copper-nickel tailings, thus proving improvements in soil properties of equal magnitude between both groups.

Biocement samples resulting from tests using CaCl_2_×H_2_O and urea in ratios of 0.5 M: 1 M (Chen et al., 2017), 0.3 M: 1 M (Meyers et al., 2011), 0.8 M: 1 M (Duan et al., 2021), were discarded because they did not maintain the shape of the mold, thus evidencing that the mine tailings were not conglomerated (data not shown). However, the samples where a 1 M: 0.8 M (calcium: urea) ratio was used, inverse to that used by Wang et al. (2022), maintained the shape of the mold, did not deform and were malleable, even though a loss of mass was found when tapped. Since the samples with the best design were those obtained by using a higher proportion of calcium with respect to urea, this could indicate that the concentration of calcium in the medium is a critical and limiting factor in the formation of biocement, in agreement with findings obtained with *S. pasteurii*. Tests conducted with this train at different concentrations of calcium, showed that biocementation with higher calcium supplementation (2.5 M CaCl_2_) significantly increased the hardness of the surface of the biocement samples (Zúñiga-Barra et al., 2023). On a microscopic scale, the selected biocement samples showed that in both biocementations with native and reference strains, bacteria colonized cracks and micropores inside the soil particles, being possible to isolate a significantly higher number of CFUs with ureolytic activity in the biocement formed with *Paenibacillus* sp. T130-13 on the contrary to the lower number of CFUs obtained in the biocement formed with *S. pasteurii* (Figures 5, 6). Additionally, these CFUs were a further time identified by sequencing their 16S ribosomal RNA gene, confirming the identity of the ureolytic bacteria (data not shown). Also in Figure 6 it was possible to identify by FE-SEM-EDS microscopy also the presence of calcium (yellow), carbon (blue) and oxygen (green) in the cell membrane of the bacteria, potentially indicating the formation of calcium carbonate and the role of nucleation proposed by Stocks-Fischer et al. (1999) (Figures 5D, E).

Bacterial biofilms with high calcium, carbon and oxygen content were also identified in a widespread form between tailing’s particles and on their surface (Figure 6). This would indicate the ability of bacteria to colonize the soil particle despite the presence of PTEs in the MT. In relation to the considerations above, the accumulation of calcium ions on the surface of the cells (Figure 7) and in the extension of the biofilms (Figure 6), agrees with results from Ercole et al. (2007) that described the potential role of the cell membrane and bacterial exudates in concentrating calcium ions that subsequently interact with the carbonates released by the cell and then form calcium carbonate. On the other hand, carbonates increase the neutralization potential (Qureshi et al., 2016), thus if bacteria by MICP synthesize carbonates then they will increase the neutralization potential, consequently improving the chemical stability of the MT and decreasing the possibility of generating acid mine drainage (AMD), therefore, in future studies it will be necessary to include acidity potential calculations, and *in situ* testing, in inactive tailings.

Despite the promise of MICP for the biocementation of MT, there are still challenges and limitations to the wide use of this technology. The most critical aspect is the release of ammonia from the ureolysis process (Erdmann and Strieth, 2023), therefore it is growing the interest in finding valuable alternatives to the use of urea and/or microorganisms that metabolize urea for biocementing MT. For example, the use of microalgae such as *Anabaena variabilis* (Zúñiga-Barra et al., 2023; Zúñiga-Barra et al., 2024) opens new interesting perspectives to this research, by reducing negative impacts and avoid ammonia emission into the environment.

## 5 Conclusion

The isolated native bacterial strains showed the ability at biocementing copper mine tailings by MICP despite the presence of PTEs and other compounds potentially toxic to microbial growth. Also, biocementation with the bacteria improved soil stabilization as demonstrated by the increased mechanical compressive strength and the decreased wind erosion, thus proving that carbonate precipitation between pores enhanced soil compaction and cohesion. These findings support the potential of native strains in biocement formation. However, the need for further and more extensive research is evident in order to optimize the different techniques employed. Aspects such as: (i) set the optimal growth parameters for the strains; (ii) establish the conditions that maintain a constant urease activity; and (iii) promote greater precipitation of calcium carbonates could be addressed. In addition, standardization of soil mechanics tests is crucial to compare the characteristics of biocement with products from the traditional cement industry.

## Data Availability

The datasets presented in this study can be found in online repositories. The names of the repository/repositories and accession number(s) can be found in the article/Supplementary material.
